# Pre-invasive compaction and spatial organization influence invasion patterns in MDA-MB-231 spheroids

**DOI:** 10.1007/s44164-026-00139-5

**Published:** 2026-07-06

**Authors:** You-shin Chen, Dulce Ciuffetelli, Meaghan Snider, Yi-Chun Chen, Katheren Barger, Chloe Horcha-Fuwell, Joseph Sucic

**Affiliations:** Department of Biology, University of Michigan-Flint, Flint, MI 48502 USA

**Keywords:** MDA-MB-231, Basement membrane extract 3D invasion assay, Triple-negative breast cancer, Tumor spheroids, Spheroid compaction

## Abstract

**Purpose:**

Cancer metastasis is the primary cause of cancer-related mortality, and this process is initiated by the invasion of tumor cells into surrounding tissues. Three-dimensional (3D) spheroid invasion assays are widely used to study tumor invasion in triple-negative breast cancer (TNBC), yet reported invasion patterns of MDA-MB-231 spheroids in basement membrane extract (BME) systems vary considerably. The physicochemical determinants underlying this variability remain poorly defined.

**Methods:**

Here, we use time-lapse z-stack imaging combined with quantitative tracking of matrix-associated landmarks to investigate how pre-invasive cellular organization, matrix composition, and spatial heterogeneity influence invasion behavior in MDA-MB-231 multicellular tumor spheroids cultured in the presence and absence of Invasion Matrix (IM).

**Results:**

Prior to invasion, peripheral cellular aggregates undergo morphological reorganization, while exploratory protrusions exhibit dynamic extension, retraction, merging, and bifurcation events that ultimately give rise to a reduced number of dominant protrusive branches. Matrix composition influences invasion-associated behavior, with reduced collagen density accelerating extracellular matrix (ECM) displacement and altering the spatial organization of spheroid-associated cells. In addition, distinct protrusion morphologies were observed at different focal planes within the same spheroid, highlighting spatial heterogeneity in invasion-associated phenotypes.

**Conclusion:**

These findings demonstrate that invasion in spheroid models is preceded by dynamic cellular and matrix-associated processes and is influenced by matrix composition and spatial heterogeneity. Together, these observations provide a framework for understanding variability in spheroid invasion assays.

**Supplementary Information:**

The online version contains supplementary material available at https://doi.org/10.1007/s44164-026-00139-5.

## Introduction

Cancer accounts for an estimated 9.7 million deaths globally in 2022, according to the International Agency for Research on Cancer (IARC) of the World Health Organization (WHO) [1]. Metastasis, the systematic dissemination of tumor cells, remains the leading cause of cancer-related mortality [2, 3]. While tumor invasion is a critical step in the metastatic cascade, the context-dependent mechanisms governing the transition from a non-invasive to an invasive state are complex and remain incompletely understood [4–6]. Further investigation is required to understand how the initial mechanical organization of the tumor mass and its reciprocal interactions with the extracellular matrix (ECM) contribute to the emergence of distinct invasion phenotypes [7, 8].

Triple-negative breast cancer (TNBC) is defined by the absence of estrogen receptor (ER), progesterone receptor (PR), and human epidermal growth factor receptor 2 (HER2) expression [9]. TNBC accounts for approximately 10–20% of all breast cancer cases and is associated with aggressive invasion, early recurrence, and a high propensity for metastatic dissemination, contributing disproportionately to breast cancer mortality [10–12]. These clinical features underscore a critical need to better understand the molecular and cellular mechanisms driving TNBC progression to develop more effective therapeutic strategies.

Three-dimensional (3D) spheroid invasion assays are widely used *in-vitro* models for studying tumor invasion, as they recapitulate key features of tumor architecture and cell-ECM interactions [13–17]. In particular, basement membrane extract (BME)-based systems provide a physiologically relevant platform for studying invasion-associated behaviors and cell–ECM interactions in TNBC cells. [18–20]. The basement membrane is a specialized ECM structure composed primarily of laminins, type IV collagen, nidogen, and heparan sulfate proteoglycans, forming an interconnected network that regulates both tissue architecture and cell–ECM interactions relevant to tumor invasion [21, 22].

The BME-based spheroid invasion assay has been widely applied to investigate the invasive behavior of the TNBC cell line MDA-MB-231 [18, 23–31]. Standardized commercial platforms, such as the Cultrex® 3D Spheroid BME Cell Invasion Assay (3500–096-K), have enabled reproducible experimental workflows. However, despite their widespread use, a consensus on the characteristic invasion phenotype of MDA-MB-231 spheroids in this system remains lacking. Previous studies have reported diverse invasion patterns, including “spider-web”-like radial dissemination with a loosely organized multicellular migratory rim surrounding the spheroid core [23, 24], dense, symmetric, radially oriented protrusions extending from a compact spheroid core [18, 25–28], asymmetric protrusive invasion from a loosely aggregated spheroid [29], and highly polarized, unidirectional invasion [30]. Importantly, the sources of this phenotypic variability remain incompletely understood. This variability complicates the interpretation of invasion assays, particularly in mechanistic studies and therapeutic evaluation.

While biochemical signaling pathways regulating invasion have been extensively investigated, the contribution of pre-invasive multicellular organization and local microenvironmental context remains less well defined. In particular, how physical organization within spheroids influences invasion dynamics and phenotypic variability has not been systematically examined.

In this study, we investigate how pre-invasive multicellular organization and physical parameters are associated with invasion behavior in MDA-MB-231 spheroids. Specifically, we examine the relationship between spheroid organization, ECM remodeling, matrix composition, and invasion-associated phenotypes using time-lapse z-stack imaging and quantitative analysis of ECM displacement. By characterizing these dynamic processes, we aim to identify factors associated with phenotypic variability in BME-based spheroid invasion assays. Improved understanding of these variables may contribute to enhanced reproducibility and more consistent interpretation of 3D TNBC invasion models.

## Materials and methods

### Cell culture

MDA-MB-231 triple-negative breast cancer cells were a generous gift from Dr. Jessica Kelt, Department of Biochemistry and Green Chemistry at University of Michigan-Flint. MDA-MB-231 cells were cultured in Dulbecco’s Modified Eagle Medium (DMEM; Gibco Cat. No. 10564–011) supplemented with sodium pyruvate (1x; Gibco Cat. No. 11360–070), non-essential amino acids (1x; Gibco Cat. No. 11140–050), 10% FBS (Gibco Cat. No. A5669701), 0.05 mg/mL gentamicin (Gibco Cat. No. 15750–060), and 0.25 μg/mL amphotericin B (Gibco Cat. No. 15290–018). MDA-MB-231 cells were maintained in a 37 ˚C cell culture incubator, supplemented with 5% CO_2_.

### Spheroid formation and 3D invasion assay

MDA-MB-231 cells were sub-cultured twice (representing at least 4 population doublings) post-thawing to minimize phenotypic variability associated with recovery from cryopreservation and to standardize culture conditions across experiments. Spheroids were generated following the manufacturer’s protocol for the Cultrex® 3D Spheroid BME Cell Invasion Assay (R&D Systems, Cat. No. 3500–096-K). Briefly, 3,000 MDA-MB-231 cells were suspended in a 50 µL master mix, consisting of 1 × Spheroid Formation Extracellular Matrix (ECM) and complete growth medium. According to the manufacturer’s instruction, Spheroid Formation ECM contains a proprietary blend of ECM proteins derived from murine Engelbreth-Holm-Swarm (EHS) sarcoma cells and is optimized for spheroid formation. The cell suspension was then seeded into each well of ultra-low attachment (ULA) 96-well round-bottom plates (Corning® 7007) and centrifuged at 200 × *g* for 3 min at 4 °C to promote initial cell aggregation.

Following a 72-h incubation at 37 °C and 5% CO_2_, 50 μL of Cultrex® Invasion Matrix (IM) was added to designated wells. IM is also a proprietary ECM mixture derived from murine EHS sarcoma cells, containing BME and collagen. After IM solidification, 100 μL of complete medium was overlaid in each well. Spheroids were then transferred to a BioTek Cytation 1 Cell Imaging Multimode Reader (Agilent) maintained at 37 ˚C and 5% CO_2_ for image acquisition.

### Z-stack time-lapse imaging

Time-lapse z-stack phase-contrast imaging was performed using BioTek Cytation 1 Cell Imaging Multimode Reader (Agilent). Images were acquired using 4 × objective lens at approximately 2 h intervals over a 54 h–92 h period under standard cell culture conditions (37 ˚C and 5% CO_2_) to monitor the kinetics of spheroid invasion into the surrounding matrix. The image acquisition intervals were determined by the time needed to capture all spheroids within the 96-well plate. For z-stack acquisition, 10 focal planes (z = 0–9) were collected at each time point with a vertical step size of 54.1 μm between adjacent planes. The lowest focal plane (z = 0) corresponded to the spheroid region nearest the Ultra-Low Attachment (ULA) substrate, whereas higher z-planes progressively sampled regions extending toward the culture medium interface.

### ECM displacement analysis

Spatial calibration for time-lapse pictures of MDA-MB-231 spheroids was established based on the 4 × objective lens and the specific parameters of the automated montage stitching process, resulting in a pixel-to-micron ratio of 0.22. To quantify the mechanical reach of the MDA-MB-231 spheroids, discrete ECM ‘landmarks’—defined as high-contrast fiber intersections or matrix granules—were identified at varying distances from the spheroid periphery. The *xy*-coordinates of these landmarks were tracked over time using ImageJ software to calculate the magnitude and velocity of centripetal displacement toward the spheroid core. The mean velocity was derived from dividing the total displacement (μm) by the elapsed time (h). All spheroids analyzed in this study were generated from independently prepared cultures and imaged under identical experimental conditions to provide biological replication. A total of 30 spheroids were initially cultured in each condition. One spheroid in the + IM group and one spheroid in the − IM group extended beyond the z-stack acquisition range and were therefore excluded from quantitative analysis. Consequently, 29 MDA-MB-231 spheroids per condition, derived from two independent experiments, were included in the final analysis. Statistical comparisons of ECM displacement velocities between the two groups were performed using an unpaired, two-tailed Student’s *t*-test, with *p*-values lower than 0.05 considered statistically significant.

## Results

To define how invasion emerges in MDA-MB-231 spheroids, we first examined pre-invasive cellular organization, followed by the development of invasive structures and their interaction with the surrounding ECM. We then assessed how matrix composition and spatial positioning contribute to heterogeneity in invasion behavior.

**Fig. 1:**
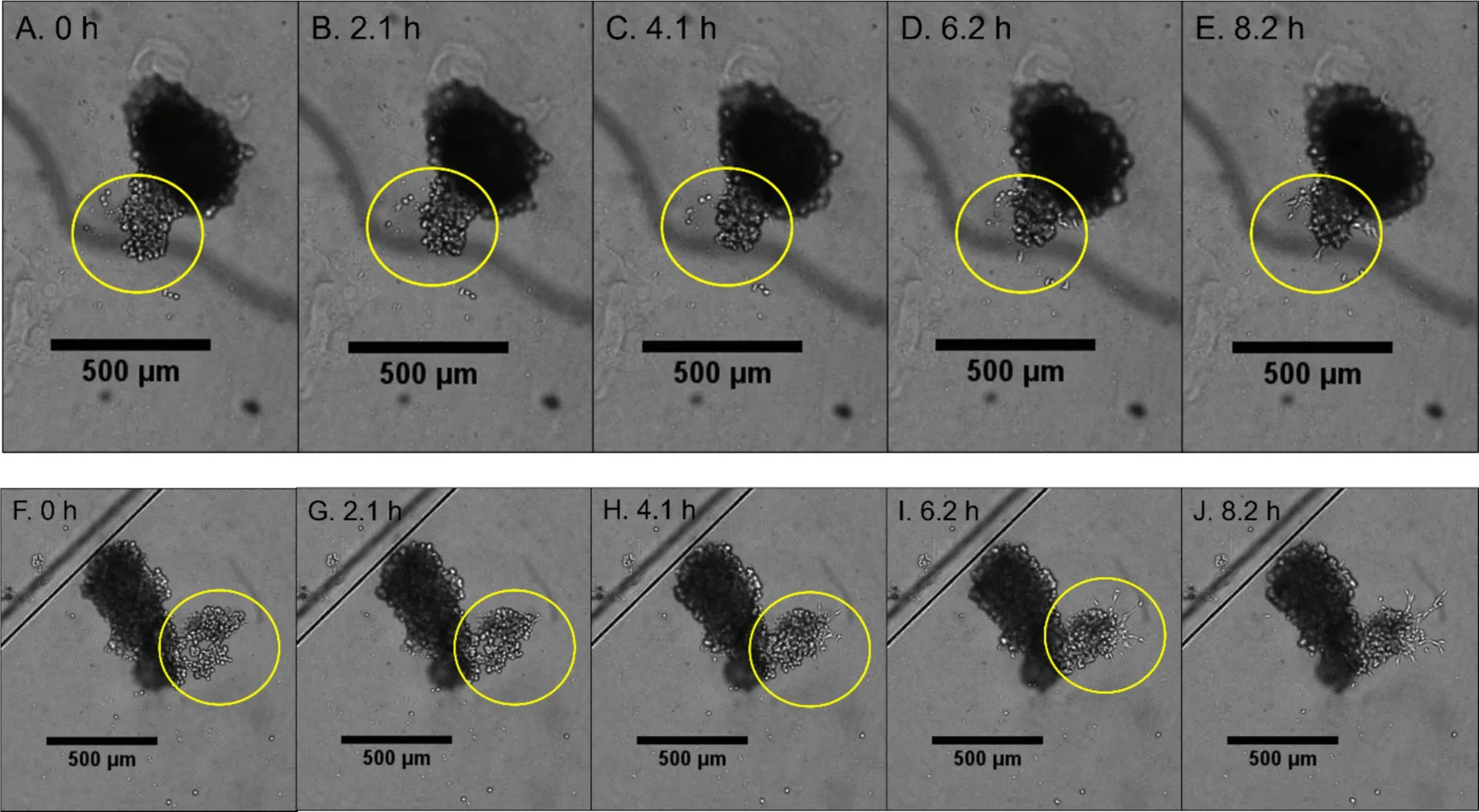
Time-lapse sequences (123 min/frame) illustrating the pre-invasive compaction of localized, peripheral cellular aggregates. (**A**–**E**) A discrete region of loosely associated cells at the inferior-left periphery (approx. 7 o’clock position; yellow circle) undergoes progressive inward reorganization toward the primary spheroid mass. (**F**–**J**) A similar compaction event is observed at the inferior-right periphery (approx. 5 o’clock position, yellow circle). In both cases, peripheral cells consolidate into a more cohesive structure prior to the emergence of invasive protrusions, suggesting structural compaction precedes coordinated invasion. Scale bar = 500 µm

**Fig. 2:**
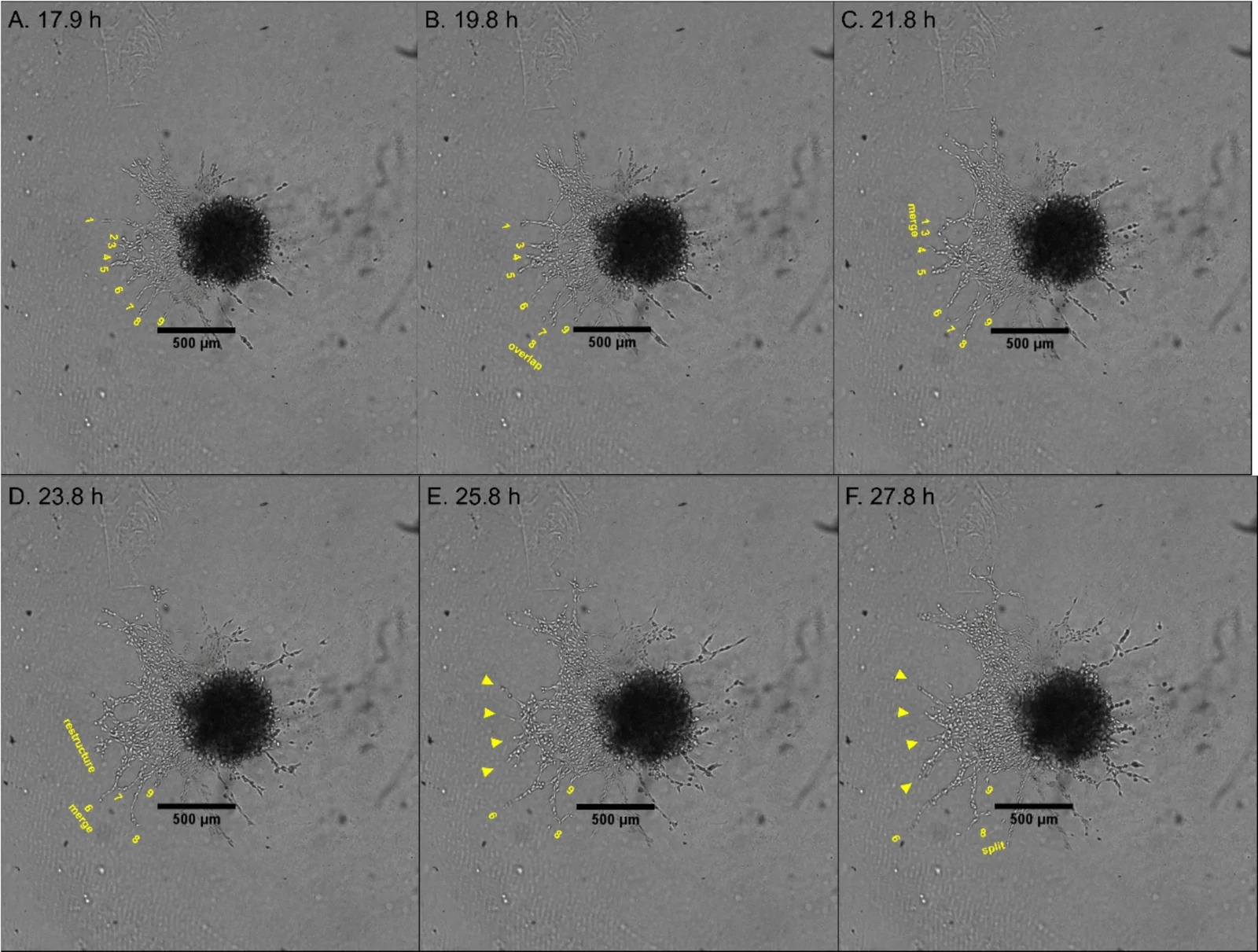
Representative phase-contrast images (119 min/frame) illustrating dynamic refinement of exploratory protrusions during invasion. (A) Multiple transient protrusions are initially visible (labeled 1–9). Over about 20 h, these protrusions undergo restructuring events, including retraction (protrusion 2; **A**–**B**), merging (protrusions 1 and 3 in B–C and protrusions 6 and 7 in **C**–**D**), and bifurcation (protrusion 8; **E**–**F**) followed by subsequent merging with an adjacent protrusion (**F**–**G**). (**D**–**L**) Progressive consolidation results in a reduced number of dominant protrusive branches (protrusions 1, 3, 4, and 5; yellow triangles). Cells not incorporated into these dominant branches appeared to undergo centripetal redistribution, forming a localized aggregate at the spheroid boundary prior to incorporation into the invasive front at the later time points shown in Video 2. Scale bar = 500 μm

**Fig. 3:**
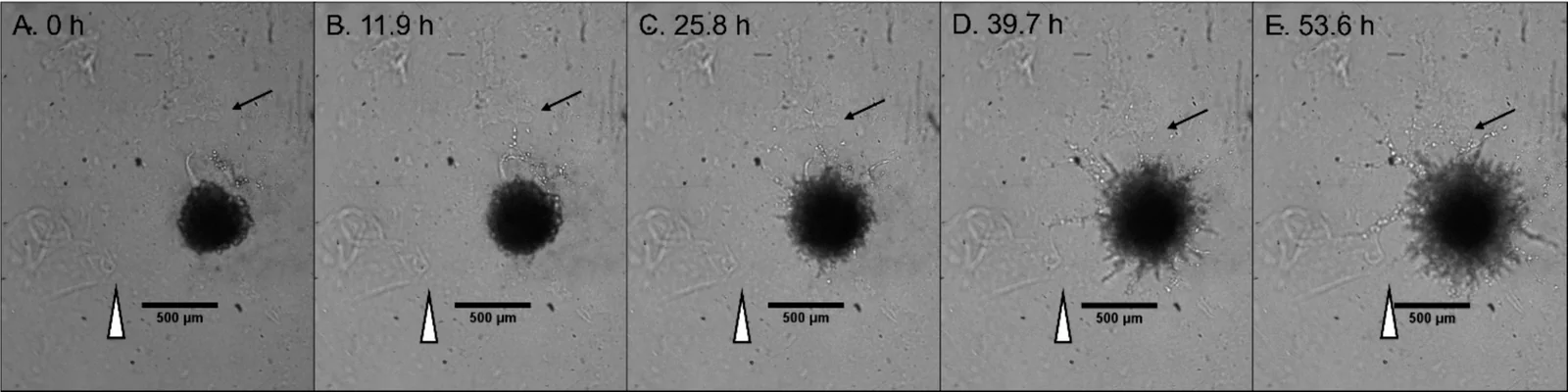
Representative micrographs illustrating centripetal ECM displacement induced by MDA-MB-231 spheroid. Matrix-associated landmarks (white triangle and black arrow) exhibit inward movement toward the spheroid core, with average velocities of 2.0 μm/h and 3.3 μm/h, respectively. Scale bar = 500 µm

**Fig. 4:**
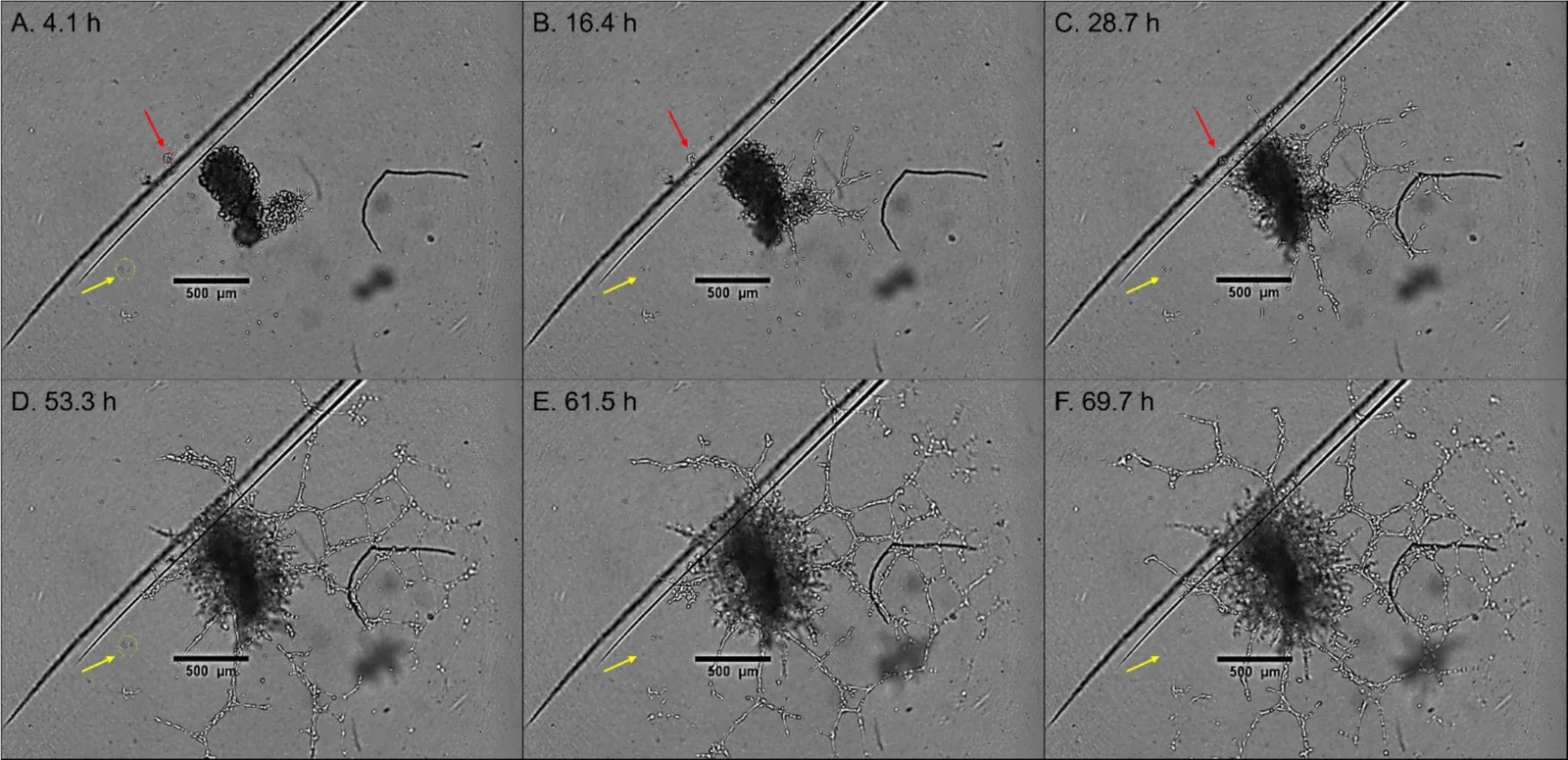
Regional association between ECM displacement and protrusion emergence. Red arrows indicate regions of early protrusive activity associated with ECM displacement toward the spheroid core, with an average velocity of 5.3 μm/h (**A**–**C**). Yellow arrows indicate a region where the matrix remained static during the early phase, with detectable ECM displacement occurring only after the emergence of invasive protrusions. Between 53.3 h and 69.7 h, ECM displacement in this region reached an average velocity of 4.3 μm/h (**A**–**F**). Scale bar = 500 µm

**Fig. 5:**
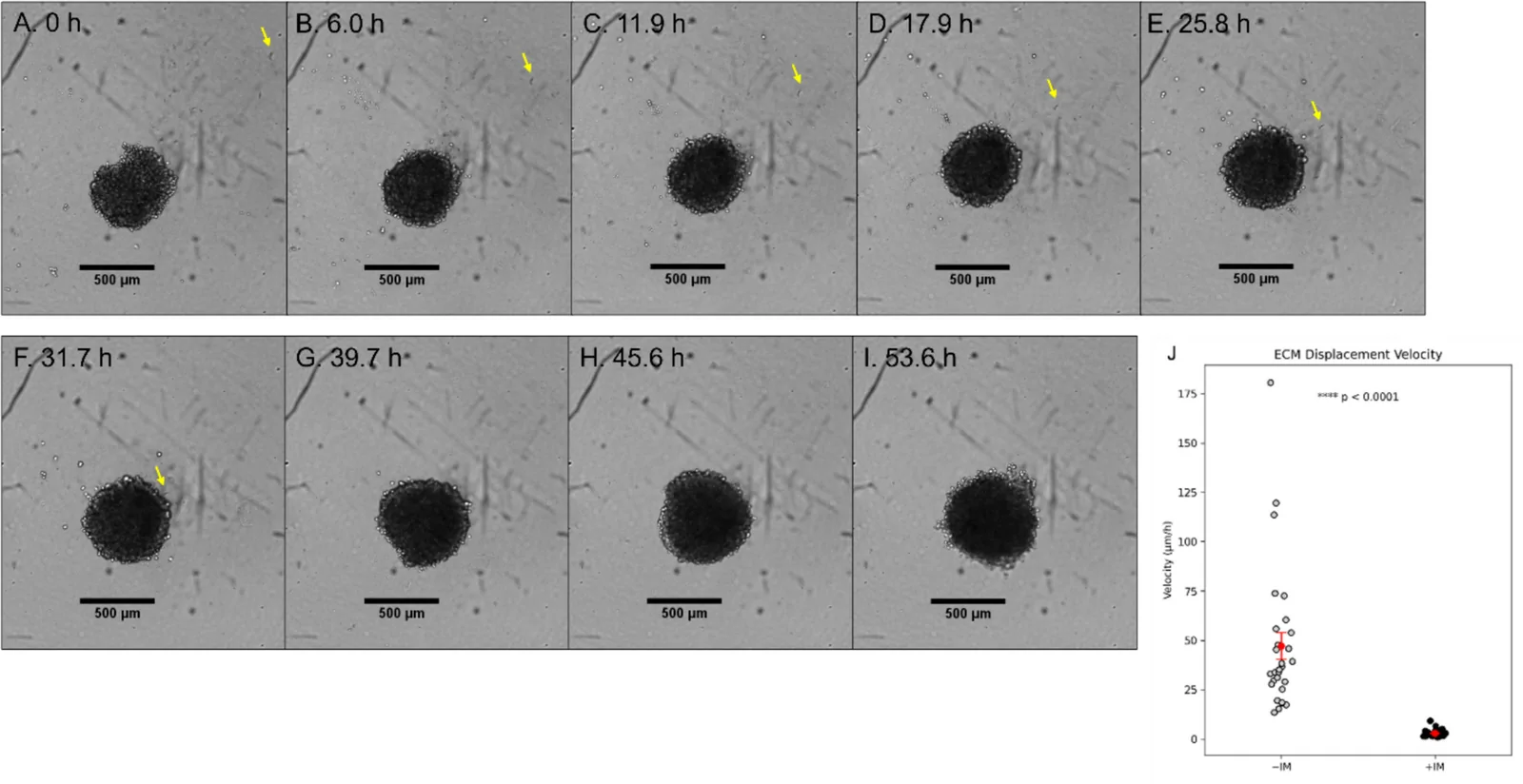
Time-lapse imaging of an MDA-MB-231 spheroid cultured in Spheroid Formation ECM without the supplementary Invasion Matrix (IM) illustrating ECM displacement. Centripetal movement of a matrix-associated landmark (yellow arrow) is observed, with an average velocity of 31 µm/h (A-F). In contrast to cohesive multicellular protrusions observed in IM-embedded conditions, cells form a peripheral halo of dispersed cellular aggregates (I). Scale bar = 500 µm. Quantification of ECM displacement velocity in spheroids cultured in the absence (− IM) or presence (+ IM) of IM. Each point represents an individual spheroid (*n* = 29 per condition, J). Markers indicate mean ± SEM. ECM displacement velocity is significantly increased in the absence of IM compared to IM-embedded conditions (unpaired two-tailed Student’s t-test, **** *p* < 0.0001)

**Fig. 6:**
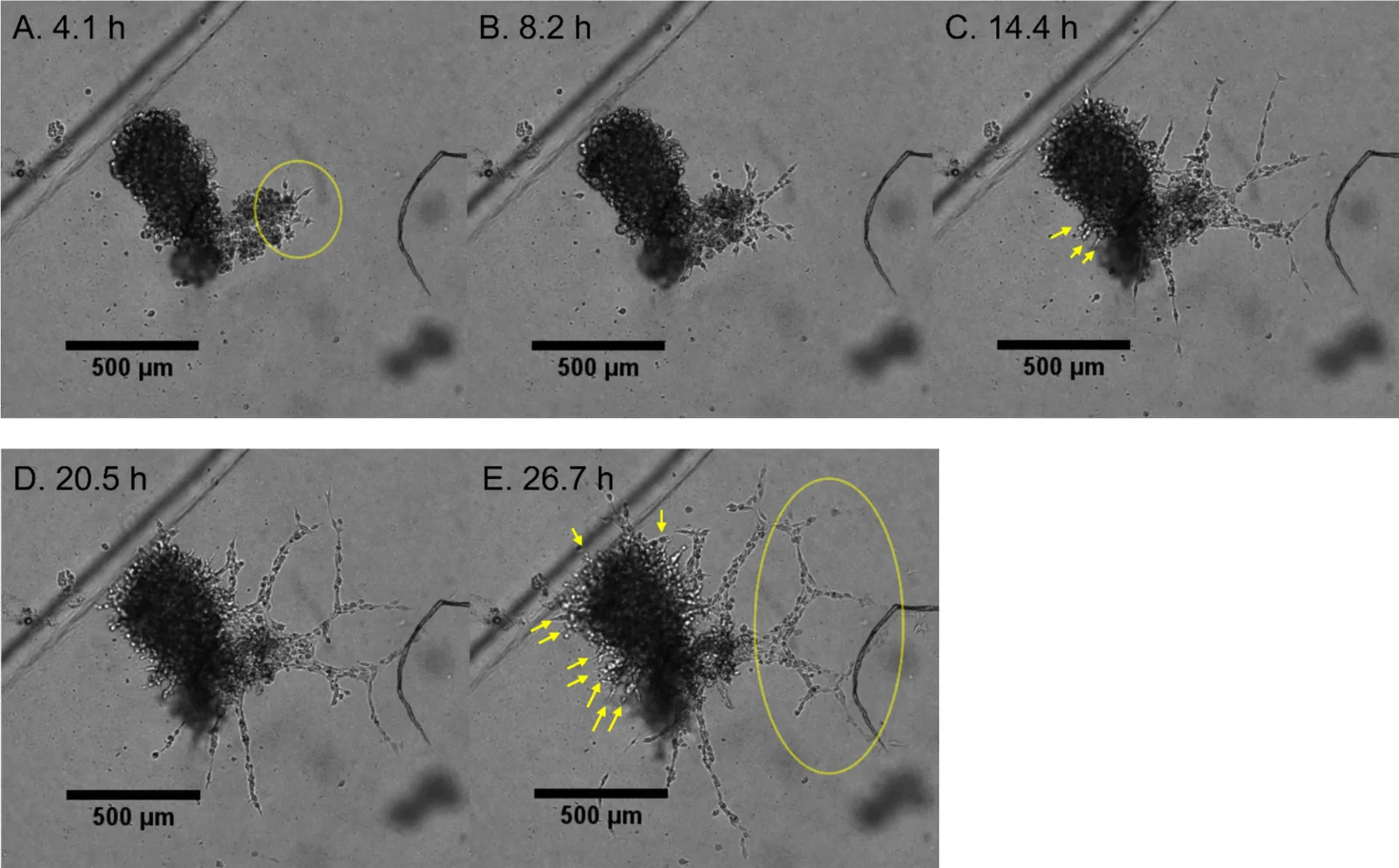
Intra-spheroid heterogeneity in the timing and architecture of invasion. (**A**) Early invasive protrusions are initiated by cells within a peripheral, loosely associated aggregate approximately 4.1 h following Invasion Matrix (IM) addition (yellow circle). (**B**) The protrusions originating from the peripheral aggregate undergo further extension and branching by 8.2 hfollowing IM addition. (**C**) In contrast, emergence of multicellular protrusive structures from the compact spheroid core is delayed, becoming apparent approximately 10 h after the onset of peripheral activity (yellow arrows). (**D**) By 20.5 h following IM addition, both the early protrusions originating from the peripheral aggregate and the laterprotrusions emerging from the compact spheroid core exhibit continued expansion and remodeling. (**E**) Distinct invasion architectures are observed within the same spheroid: the compact core exhibits radially oriented protrusions (yellow arrows), whereas the peripheral aggregate displays a disorganized, “spider-web”-like morphology characterized by lateral spreading across the basal interface

**Fig. 7:**
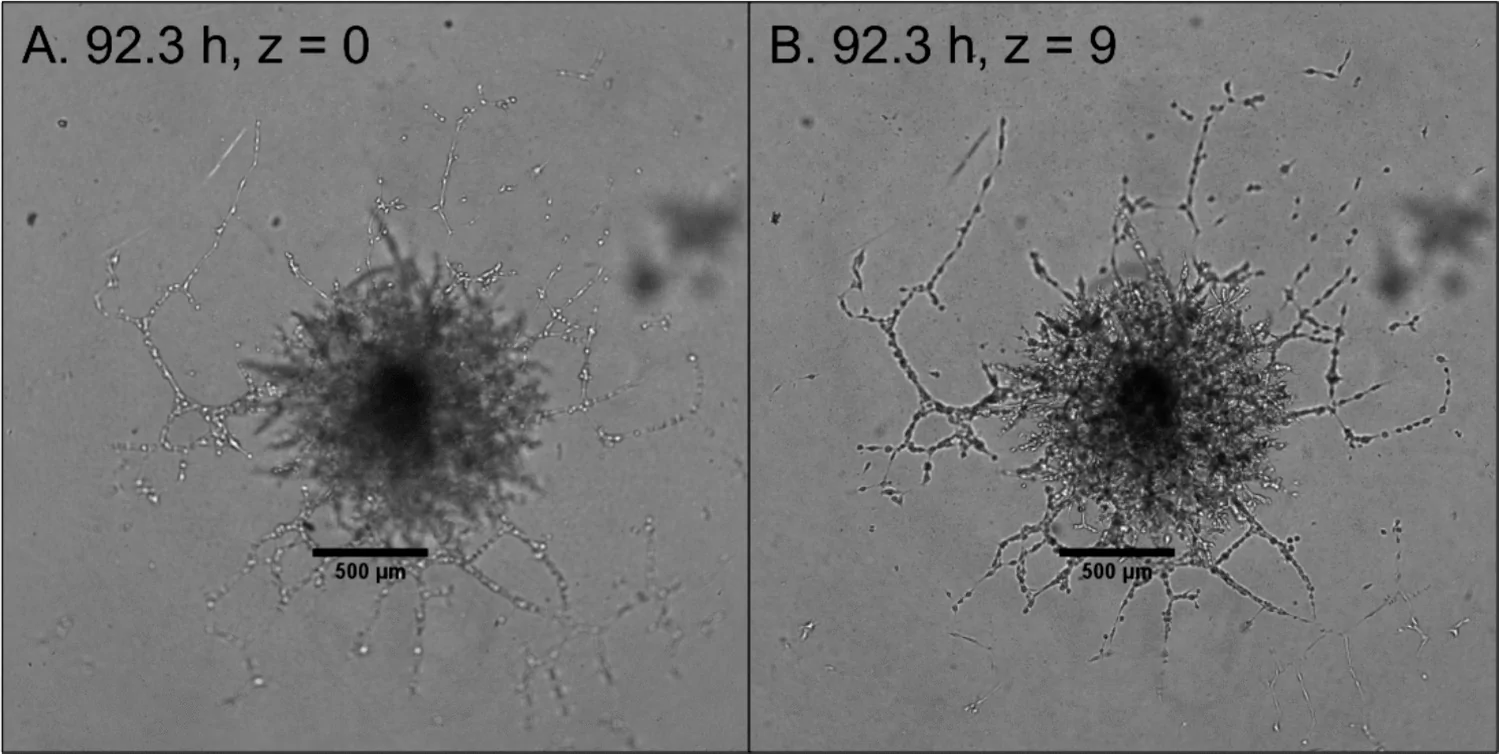
Representative phase-contrast images of MDA-MB-231 spheroids captured at distinct focal planes (z-positions) following 94 h of invasion. (**A**) Lower focal plane (*z* = 0): The spheroid region near the Ultra-Low Attachment (ULA) substrate exhibits broad cellular spreading with a more “spider-web”-like planar morphology. The protrusions in focus appear as laterally distributed cellular extensions. The apparent brightness of these structures arises from phase-contrast–induced edge enhancement, which amplifies contrast in thin cellular extensions. (**B**) Upper focal plane (*z* = 9): In contrast, protrusions in focus exhibit elongated, radially oriented multicellular extensions extending away from the spheroid core. Lower-plane protrusions remain visible but are out of focus at this focal plane due to their separation along the z-axis. Phase-contrast imaging enhances the visibility of the in-focus protrusions by accentuating the edges of these thicker multicellular structures. These observations illustrate distinct protrusion morphologies observed at different focal planes within the same spheroid. Scale bar = 500 µm

1. Pre-invasive compaction of peripheral aggregates precedes the formation of cohesive invasive fronts
  During the Spheroid BME 3D Invasion Assays, peripheral cellular aggregates were observed in 30% of spheroids and exhibited a distinct pre-invasive reorganization phase (9/29; Fig. 1). The origin of the peripheral cellular aggregates remains unclear, as the observational nature of the imaging data does not permit definitive determination of whether these structures emerged from the primary spheroid or resulted from the convergence of neighboring cellular clusters. Initially, peripheral aggregates displayed a dispersed morphology characterized by irregular boundaries. (Fig. 1A and F). Over time, these regions underwent progressive compaction, shown by a reduction in aggregate area and the formation of a more densely packed structure (Fig. 1A–C and Fig. 1F–G). Aggregate compaction was observed in all spheroids containing peripheral aggregates (9/9).
  This compaction phase consistently preceded the emergence of invasive protrusions (Fig. 1D and H). In spheroids containing peripheral cellular aggregates, regions that underwent compaction were subsequently associated with the formation and continued remodeling of invasive protrusions. Although Fig. 1 highlights an 8.2-h interval to illustrate the compaction process, these structures were followed throughout the remainder of the 92-h imaging period and continued to exhibit invasive behavior. (Video S1–S2). These observations identify compaction as a prominent pre-invasive organizational transition that consistently precedes the emergence of coordinated invasion in this model.
  Following this compaction phase, we next examined how invasive structures emerge from these reorganized cell populations.
2. Dynamic remodeling of protrusive structures during invasion
  Early during invasion, multiple transient protrusions extended from the spheroid surface (Fig. 2 and Video 2). Over time (~ 20 h), these protrusions underwent dynamic remodeling, including extension, retraction, merging, and bifurcation events, resulting in a reduced number of dominant protrusive structures (Fig. 2).
  Cells within regions outside the dominant protrusive branches appeared to undergo centripetal redistribution, resulting in the formation of localized cellular aggregates at the spheroid periphery. These aggregates subsequently became spatially associated with the expanding protrusive structures.
  Although Fig. 2 highlights a representative 20-h interval to illustrate the remodeling process, the spheroids were continuously monitored for a total of 55 h. Throughout this period, protrusive structures continued to exhibit dynamic remodeling and invasive behavior (Video S3).
  Together, these observations indicate that invasion is accompanied by progressive remodeling of protrusive structures rather than simultaneous persistence of all initially formed protrusions.
  Given the emergence of dominant protrusive structures, we next investigated how these cellular structures interact with the surrounding extracellular matrix.
3. MDA-MB-231 spheroids induce long-range centripetal ECM displacement
  Time-lapse imaging revealed progressive centripetal displacement of the surrounding ECM, consistent with the contractile activity of MDA-MB-231 spheroids (Fig. 3 and Video 3).
  To quantify this interaction, we analyzed matrix deformation during the initial phase of spheroid-ECM contact. MDA-MB-231 spheroids induced long-range centripetal ECM displacement, with detectable movement observed at distances exceeding 500 µm from the spheroid periphery. Quantitative tracking of matrix-associated landmarks (white triangle and black arrow) revealed sustained inward movement toward the spheroid core, with average velocities of 2.0 μm/h and 3.3 μm/h, respectively (Fig. 3 and Video 3). Consistent with these representative measurements, analysis of additional spheroids (*n* = 29 total; Fig. 5J) yielded a mean ECM displacement velocity of 2.9 µm/h. These observations indicate that spheroids generate a spatially extensive field of ECM deformation during invasion.
  To further resolve the relationship between invasion and matrix remodeling, we next analyzed the spatial and temporal coordination between protrusion formation and ECM displacement.
4. ECM Displacement occurs in regions exhibiting protrusion emergence
  Time-lapse imaging revealed regional and temporal differences in ECM displacement during invasion (Fig. 4 and Video 4). Regions in which protrusive branches appeared earlier also exhibited earlier matrix displacement, whereas areas lacking protrusions remained largely static during the initial phase of observation.
  For example, the matrix-associated landmark indicated by the yellow arrow in Fig. 4 remained stationary for approximately the first half of the recording period (~ 50 h), with detectable displacement toward the spheroid core becoming apparent only during the later phase of the sequence, following protrusion emergence in that region. In contrast, the landmark indicated by the red arrow exhibited inward displacement within the first 5 h in a region displaying early protrusive activity.
  These observations indicate that ECM displacement was not uniformly distributed around the spheroid but instead exhibits pronounced regional heterogeneity. In addition, ECM displacement was observed in regions exhibiting protrusion emergence, whereas little displacement was detected in regions lacking protrusions during the early phase of imaging. Together, these findings suggest that spheroid-induced matrix deformation develops in a spatially heterogeneous manner during invasion.
  Because ECM displacement was observed in regions exhibiting invasion-associated protrusions, we next examined how matrix composition influences these processes. The Invasion Matrix contains additional collagen relative to the Spheroid Formation ECM and therefore provides a distinct microenvironment for cell–matrix interactions. By comparing spheroids cultured in the presence and absence of IM, we sought to determine whether changes in matrix composition are associated with differences in ECM displacement behavior and invasion morphology.
5. Matrix composition modulates ECM displacement dynamics and invasion mode
  To investigate the influence of matrix composition on spheroid-induced ECM displacement, we analyzed spheroids cultured in the absence of the supplementary Invasion Matrix (IM), using only the Spheroid Formation ECM. Under these conditions, spheroids continued to induce long-range centripetal ECM displacement (Fig. 5 and Video 5), consistent with sustained contractile activity.
  However, the kinetics of matrix displacement were markedly altered. Tracking of a matrix-associated landmark (Fig. 5A–F, yellow arrow) revealed an average displacement velocity of 31 µm/h, representing an approximately ten-fold increase compared to spheroids embedded in IM. Across all analyzed spheroids (*n* = 29), the mean ECM displacement velocity in the absence of IM was 47.1 µm/h (Fig. 5J). While individual spheroids exhibited variability in displacement velocity, values were consistently elevated relative to IM-embedded conditions, likely reflecting heterogeneity in local cellular organization and microenvironmental context.
  Along with this accelerated matrix movement, the mode of cellular invasion differed substantially. In IM-embedded conditions, spheroids formed cohesive, multicellular invasive protrusions. In contrast, spheroids cultured without IM failed to generate consolidated invasive fronts. Instead, cells progressively dissociated from the spheroid core, forming a peripheral halo of dispersed cellular aggregates by the end of the observation period.
  To further compare invasion behavior between matrix conditions, the total area occupied by spheroid-associated cells was quantified at the conclusion of the imaging period. Spheroids cultured in the presence of IM exhibited significantly larger occupied areas than spheroids cultured in the absence of IM (*n* = 29 per condition, Student’s *t*-test, *p* < 0.001; Figure S1). On average, spheroids embedded in IM occupied approximately 60% greater area than spheroids cultured without IM. These findings support the observation that matrix composition influences the spatial extent and organization of spheroid-associated cells during invasion. This finding is consistent with previous reports showing that variations in ECM composition and collagen-dependent matrix architecture can regulate tumor cell migration, invasion morphology, and collective invasion behavior in three-dimensional microenvironments [22].
  These findings indicate that while ECM displacement is maintained in the absence of IM, matrix composition strongly influences the organization of invasion. Reduced matrix density was associated with increased ECM displacement velocity and altered invasion morphology relative to IM-embedded spheroids. In contrast to the protrusive structures observed in IM-containing cultures, spheroids cultured without IM exhibited dispersed cellular aggregates surrounding the spheroid core and occupied a significantly smaller area. This finding is consistent with previous reports demonstrating that ECM composition and collagen-dependent matrix architecture can influence tumor cell invasion, migration patterns, and multicellular organization within three-dimensional culture systems. Together, these findings indicate that matrix composition influences both ECM deformation dynamics and the spatial organization of spheroid-associated cells.
6. Intra-spheroid heterogeneity reveals temporal and structural diversity in invasive behavior
  While matrix composition influenced invasion behavior, we also observed substantial variability in invasion patterns within individual spheroids under the same ECM conditions, suggesting that additional factors contribute to this heterogeneity.
  Time-lapse imaging revealed pronounced intra-spheroid heterogeneity in both the timing and morphology of invasive behavior (Fig. 6). Distinct differences were observed between peripheral cell populations and the compact spheroid core.
  In the representative spheroid shown in Fig. 6, cells within peripheral, loosely associated aggregates initiated exploratory protrusions as early as 4.1 h following the addition of Invasion Matrix. In contrast, the compact spheroid core remained largely quiescent during this early phase (yellow circle; Fig. 6A), with multicellular protrusion structures emerging approximately 10 h later (yellow arrows; Fig. 6C). A similar temporal pattern, in which invasion emerged first from peripheral aggregates before becoming evident in the compact core, was observed in all spheroids exhibiting both morphologies, although the precise delay was not quantified across the full population.
  In addition to temporal differences, the architecture of invasion varied between these regions. The compact core generated persistent, radially oriented multicellular protrusions extending outward from the spheroid core. Conversely, peripheral aggregates exhibited a more disorganized network of extensions, forming a “spider-web”-like morphology with lateral spreading across the basal interface (Fig. 6E).
  These observations indicate that invasive behavior within a single spheroid is spatially heterogeneous, with distinct cellular subpopulations exhibiting different invasion dynamics and structural organization. The degree of aggregate compaction and spatial positioning within the spheroid appear to be associated with both the timing of invasion onset and the resulting invasive architecture.
7. Distinct Invasion Morphologies Observed at Different Focal Planes
  Beyond radial heterogeneity, we next examined whether invasion morphology differed across focal planes along the z-axis within the same spheroid. Time-lapse imaging revealed pronounced spatial asymmetry in protrusion morphology at different z-positions within MDA-MB-231 spheroids (Fig. 7 and Video 6). Distinct protrusive morphologies were observed at the upper focal planes compared to the lower focal planes.

At the upper focal plane (*z* = 9), spheroids exhibited elongated, radially oriented multicellular protrusions extending away from the spheroid core. In contrast, the lower focal plane (*z* = 0) displayed broader cellular spreading with a more planar, “spider-web”-like morphology. These distinct morphologies were observed within the same spheroid but at different focal planes along the z-axis. Similar differences in protrusion morphology across focal planes were observed in other spheroids under the same experimental condition; however, the present study did not include population-level quantification of these morphological patterns.

The observed differences are consistent with spatial heterogeneity in the local microenvironment surrounding the spheroid. The lower focal planes are located nearer the Ultra-Low Attachment (ULA) substrate, whereas the upper focal planes are positioned closer to the overlying matrix and culture medium. However, the present imaging approach does not permit definitive determination of the precise three-dimensional positioning of individual protrusions or the extent to which local matrix–substrate interactions contribute to the observed morphologies. Therefore, these observations should be interpreted as descriptive differences in protrusion morphology observed across focal planes rather than definitive evidence of distinct invasion mechanisms. Representative examples are shown in Fig. 7.

Together, these results demonstrate that invasion in MDA-MB-231 spheroids is preceded by a series of dynamic cellular and matrix-associated events rather than appearing as an immediate phenotype. Peripheral cellular aggregates adjacent to the spheroid core undergo morphological reorganization prior to the emergence of protrusive structures, followed by a phase in which multiple transient protrusions exhibit extension, retraction, merging, and bifurcation events. Over time, these dynamic behaviors give rise to a reduced number of dominant protrusive branches. Concurrently, ECM displacement is observed in regions exhibiting protrusion emergence, indicating spatial heterogeneity in matrix deformation during invasion. Matrix composition influences both ECM displacement dynamics and the spatial organization of spheroid-associated cells, while distinct protrusion morphologies are observed at different focal planes within the same spheroid. Collectively, these findings highlight the contributions of cellular organization, matrix interactions, and spatial heterogeneity to invasion-associated phenotypes in 3D spheroid models and provide a framework for understanding variability in spheroid invasion assays.

## Discussion

Three-dimensional spheroid invasion assays are widely used to model tumor invasion; however, variability in reported invasion patterns has limited their reproducibility and interpretation [18, 23–31]. Our findings suggest that this variability may arise, in part, from differences in pre-invasive spheroid compaction level, spheroid vertical location relative to the cell culture well, and ECM composition, rather than solely from intrinsic differences in cellular invasive capacity. By characterizing pre-invasive states and the corresponding invasion outcomes, this work provides a basis for interpreting phenotypic diversity within standardized BME-based spheroid systems.

The pre-invasive compaction observed in peripheral cellular aggregates is consistent with collective invasion dynamics observed in tumors. In carcinoma systems, collective invasion is associated with the maintenance of cell–cell adhesion and actomyosin-mediated contractility, which promote cohesive group behavior and collective force generation [32, 33]. Such compaction processes are likely to enhance cellular cohesion within tumor clusters, facilitating coordinated movement and more effective transmission of forces across the invading front.

Following compaction of loose cell aggregate toward the compact spheroid, we observed a dynamic phase of exploratory protrusion formation and refinement. The presence of multiple transient protrusions, followed by their consolidation into a smaller number of persistent invasive branches, suggests a selection-like process in which only a subset of protrusions become stabilized. This behavior is consistent with previous descriptions of collective invasion, where leader-cell–guided protrusions coordinate directional movement while less stable extensions retract [32, 33]. Such dynamic refinement may enable spheroids to efficiently probe the surrounding matrix and establish mechanically favorable paths for invasion.

The temporal relationship between compaction and subsequent protrusion stabilization suggests that multicellular organization may contribute to the emergence of coordinated invasion behavior. However, although compaction consistently preceded the development of invasive structures in the spheroids examined here, the present study does not necessarily establish a causal role for compaction in invasion initiation. Future studies incorporating perturbations of cell–cell adhesion, actomyosin contractility, or spheroid organization will be required to determine whether compaction is mechanistically involved in the formation of persistent invasive fronts.

Our observations further demonstrate that ECM displacement is spatially and temporally associated with protrusion formation, supporting a close coupling between cellular invasion and matrix deformation. The presence of long-range, centripetal ECM displacement suggests that spheroids generate a distributed field of mechanical influence that extends beyond the immediate invasive front [34, 35]. Notably, ECM displacement was not uniform but instead emerged locally in regions where protrusions formed, indicating that matrix deformation is coordinated with invasion dynamics rather than occurring as a global, synchronous process.

Importantly, our results reveal that matrix composition modulates invasion behavior independently of the magnitude of ECM displacement. In the absence of a dense collagen-containing invasion matrix, spheroids exhibited increased ECM displacement velocity but failed to generate coordinated collective invasion, instead adopting a more dispersed cell aggregate around spheroid periphery. These findings suggest that matrix architecture provides structural constraints necessary for organizing collective behavior, rather than simply resisting cellular forces [22, 36, 37]. This distinction highlights the importance of matrix context in determining invasion mode and may help explain variability across different spheroid invasion systems.

Finally, we observed substantial intra-spheroid heterogeneity in invasion timing and morphology along both the radial and apical–basal axes. Along the radial axis, peripheral, loosely associated cell aggregates initiated earlier but less coordinated invasion, whereas compact spheroid core regions exhibited delayed but more organized collective behavior. Along the apical-basal axis, cells at the basal interface exhibited lateral spreading, forming “spider-web”-like protrusions, in contrast to radially oriented protrusions at the apical surface. These findings indicate that invasion behavior is not uniform within a spheroid but is instead shaped by local cellular organization and microenvironmental context, including spatial positioning, mechanical constraints, and boundary conditions [38–40].

Several limitations should be noted. First, while ECM displacement was quantified through the measurement of ECM landmark movement, direct measurements of cellular forces were not performed, as the current imaging-based experimental framework does not incorporate traction force or force-sensing methodologies. In addition, while the observed differences between matrix conditions are consistent with established effects of ECM composition on tumor invasion, the proprietary nature of the commercial matrices used in this study precludes identification of the specific matrix components responsible for these effects. The IM contains BME and collagen, whereas the Spheroid Formation ECM consists of a proprietary EHS-derived protein mixture optimized for spheroid formation. Consequently, the present study cannot determine which specific biochemical or structural matrix features drive the observed differences in invasion behavior.

Second, this study was conducted using a single cell line within a specific BME-based invasion assay system. While MDA-MB-231 cells represent a widely used model of aggressive breast cancer invasion, additional studies incorporating other breast cancer subtypes and tumor models will be necessary to determine the extent to which the pre-invasive organizational dynamics and invasion phenotypes described here are conserved across distinct cellular and microenvironmental contexts.

Third, although protrusive structures were observed exclusively in IM-embedded spheroids and evaluated using z-stack phase-contrast imaging, the present approach does not provide sufficient spatial resolution to unequivocally distinguish matrix invasion from migration occurring at the matrix–substrate interface. Consequently, contributions from local matrix–substrate interactions cannot be completely excluded. Future studies employing alternative matrix configurations, including approaches designed to further separate spheroids from the underlying substrate, will be valuable for determining the extent to which matrix–substrate interactions contribute to the observed protrusion morphologies and invasion-associated behaviors. Furthermore, although multicellular protrusive structures were observed throughout the invasion process, the present study did not include molecular characterization of cytoskeletal organization or cell–cell junctions. Consequently, the collective nature of these structures cannot be definitively established from the current imaging data alone. Future studies incorporating phalloidin staining and cell–cell junction markers will be valuable for further characterizing the organizational state of these invasion phenotypes.

Finally, although time-lapse phase-contrast imaging provides insight into dynamic invasion behavior, the observational nature of the present study does not establish causal relationships between compaction, ECM remodeling, and invasion. Future studies integrating molecular, biophysical, and perturbation-based approaches will be necessary to elucidate the mechanisms underlying these pre-invasive processes.

Together, these findings suggest that variability in spheroid invasion assays may arise from differences in pre-invasive cellular organization and microenvironmental context, rather than solely from intrinsic differences in cellular invasiveness. By identifying the roles of compaction, protrusion dynamics, and matrix architecture, this study establishes a framework for understanding how distinct invasion phenotypes emerge in BME-based 3D spheroid models. These insights provide guidance for improving the interpretation, reproducibility, and experimental design of in vitro 3D invasion assays, and highlight the importance of integrating mechanical and spatial parameters into future studies.

## Conclusion

In summary, this study demonstrates that invasion of MDA-MB-231 spheroids is not an immediate phenotype but emerges through a sequence of pre-invasive cellular and physical processes, including compaction, protrusion refinement, and matrix interaction. These findings establish that invasion patterns are shaped by the interplay between cellular organization, extracellular matrix context, and spatial positioning within the spheroid. By identifying these pre-invasive determinants, this work provides a framework for interpreting variability in three-dimensional spheroid invasion assays and highlights the importance of considering both mechanical and spatial factors in the design and standardization of in vitro tumor models.

## Supplementary Information

Below is the link to the electronic supplementary material.

Supplementary file1 (MP4 5138 KB) Video 1 Time-lapse sequence illustrating the directional compaction of a peripheral cellular aggregate toward the spheroid core. Images were acquired at 123 min/frame and are displayed at 1 frame/sec. Initially, peripheral cells exhibit a dispersed morphology at the inferior-left pole. Over the observation period, these cells undergo progressive inward reorganization toward the primary spheroid mass, resulting in a more cohesive structure prior to the onset of invasion. Scale bar = 500 µm

Supplementary file2 (MP4 24065 KB) Video 2 Time-lapse sequence corresponding to Figure 2, illustrating the transition from exploratory protrusions to collective invasion. Labeled exploratory protrusions (1–9) exhibit stochastic sampling, followed by dynamic merging, retraction, and bifurcation events over a 20-hour period (frames 10–21). These processes result in the emergence of a reduced number of dominant protrusive branches (yellow triangles). Concurrently, cells not incorporated into dominant branches undergo centripetal redistribution, forming a localized aggregate at the spheroid–matrix interface prior to incorporation into the invasive front. Images were acquired at 119 min/frame

Supplementary file3 (MP4 13889 KB) Video 3 Time-lapse sequence corresponding to Figure 3 (119 min/frame, played at 1 fps) illustrating long-range centripetal ECM displacement over a 92-hour period. A lateral ECM displacement event is visible within the first 10 frames (black arrow), while a displacement event at the inferior region becomes prominent during the final 10 frames (white triangle)

Supplementary file4 (MP4 27891 KB) Video 4 Time-lapse sequence highlighting regional differences in ECM displacement relative to protrusion emergence within a defined region of interest (ROI). Images were acquired at 119 min/frame and are displayed at 1 frame/sec. The matrix-associated landmark indicated by the yellow arrow remains stationary for the first half of the sequence (approx. 25 frames), corresponding to a region lacking early protrusion activity. Inward displacement of this landmark becomes detectable only during later stages, coinciding with the emergence of invasive protrusions. In contrast, the red arrow highlights a region exhibiting early ECM displacement associated with protrusive activity present at the onset of imaging. Scale bar = 500 µm

Supplementary file5 (MP4 16003 KB) Video 5 Time-lapse sequence of an MDA-MB-231 spheroid cultured in Spheroid Formation ECM in the absence of the supplementary Invasion Matrix (IM). Images were acquired at 119 min/frame and played back at 1 frame/second. Despite reduced matrix density, centripetal ECM displacement is maintained, as evidenced by the rapid inward movement of a matrix-associated landmark (yellow arrow). Quantitative tracking of this landmark reveals an average displacement velocity of 31 µm/h, nearly ten-fold faster than in IM-embedded conditions. In contrast to cohesive multicellular protrusions observed in IM conditions, cells progressively develop a peripheral halo of dispersed cellular aggregate by the end of the 53.6-hour recording period. Scale bar = 500 µm

Supplementary file6 (MP4 3591 KB) Video 6 Z-plane–resolved visualization of invasion morphologies in a MDA-MB-231 spheroid. Sequential focal planes (z = 0 to z = 9) are shown following 94 h of invasion to illustrate spatial heterogeneity across the imaging volume. Lower focal planes (z = 0) exhibit laterally distributed, “spider-web”-like cellular extensions, whereas upper focal planes (z = 9) display elongated, radially oriented multicellular protrusions extending away from the spheroid core. Intermediate z-positions demonstrate the gradual transition between these morphologies. This multi-plane visualization facilitates identification of protrusions at different focal planes and highlights the spatial separation of distinct protrusion morphologies within the same spheroid. Scale bar = 500 µm

Supplementary file7 (DOCX 104 KB)

Supplementary file8 (DOCX 26103 KB)

Supplementary file9 (AVI 9477 KB)

Supplementary file10 (AVI 9216 KB)

Supplementary file11 (AVI 7739 KB)

## Data Availability

No datasets were generated or analysed during the current study.

## Abbreviations

3D: Three dimensional
TNBC: Triple-negative breast cancer
BME: Basement membrane extract
IM: Invasion Matrix
ECM: Extracellular matrix
IARC: International Agency for Research on Cancer
ER: Estrogen receptor
PR: Progesterone receptor
HER2: Epidermal growth factor receptor 2
DMEM: Dulbecco’s Modified Eagle Medium
ULA: Ultra-low attachment
ROI: Region of interest
SEM: Standard error of the mean
