# Supplementary material for: Pre-invasive compaction and spatial organization influence invasion patterns in MDA-MB-231 spheroids
Source: In Vitro Model. 2026 Jul 6;5(4):645–57. doi: 10.1007/s44164-026-00139-5 (PMC13768457; doi:10.1007/s44164-026-00139-5)
Supplement: Supplementary file 1 [file 44164_2026_139_MOESM7_ESM.docx]

Figure S1. Quantification of the area occupied by spheroid-associated cells following invasion in the presence or absence of Invasion Matrix (IM). Individual points represent independent spheroids (n = 29 per condition). Horizontal bars indicate mean ± SEM. IM-embedded spheroids occupied significantly larger areas than spheroids cultured without IM (Student's t-test, ***p < 0.001), with an approximately 60% increase in occupied area.
