# Supplementary material for: Pre-invasive compaction and spatial organization influence invasion patterns in MDA-MB-231 spheroids
Source: In Vitro Model. 2026 Jul 6;5(4):645–57. doi: 10.1007/s44164-026-00139-5 (PMC13768457; doi:10.1007/s44164-026-00139-5)
Supplement: Supplementary file 2 [file 44164_2026_139_MOESM8_ESM.docx]

Supplemental Videos

Video S1. Time-lapse sequence corresponding to the spheroid shown in Figure 1A–E. Images were acquired over a 92-hour period under standard invasion assay conditions. The video illustrates the progressive compaction of a peripheral cellular aggregate adjacent to the spheroid core, followed by the emergence and continued remodeling of protrusive structures during the remainder of the imaging period. Figure 1 highlights an 8.2-hour interval selected to illustrate the compaction process; however, the full time-lapse sequence demonstrates that protrusion-associated activity persists throughout the duration of the experiment. Images were acquired at 123 min/frame.

Video S2. Time-lapse sequence corresponding to the spheroid shown in Figure 1F–J. Images were acquired over a 92-hour period under standard invasion assay conditions. Similar to Video S1, a peripheral cellular aggregate undergoes progressive compaction prior to the emergence of protrusive structures. The video further demonstrates that these structures continue to exhibit dynamic remodeling and invasion-associated behavior throughout the remainder of the imaging period. Figure 1 presents an 8.2-hour interval to highlight the compaction phase, whereas the full video provides the complete 92-hour observation period. Images were acquired at 123 min/frame.

Video S3. Extended time-lapse sequence corresponding to the spheroid shown in Figure 2. Images were acquired over a 55-hour period under standard invasion assay conditions. During the early phase of invasion, multiple protrusive structures emerge from the spheroid surface and undergo dynamic remodeling, including extension, retraction, merging, and bifurcation events. Over time, these processes result in the emergence of a reduced number of dominant protrusive structures. Cells located outside these dominant protrusions appear to undergo centripetal redistribution, leading to the formation of localized cellular aggregates that subsequently become spatially associated with expanding protrusive structures. While Figure 2 highlights a representative 20-hour interval to illustrate the remodeling process, the full time-lapse sequence demonstrates that protrusive structures continue to undergo dynamic remodeling and invasion-associated behavior throughout the 55-hour imaging period. Images were acquired at 119 min/frame.
